# Relationship between Volitional and Non-Volitional Quadriceps Muscle Endurance in Patients with Chronic Obstructive Pulmonary Disease

**DOI:** 10.3390/diagnostics14020190

**Published:** 2024-01-15

**Authors:** Anouk A. F. Stoffels, Neeltje A. E. Allard, Martijn A. Spruit, Peter Klijn, Maria T. E. Hopman, Roy Meys, Frits M. E. Franssen, Silvie Timmers, Bram van den Borst, Hieronymus W. H. van Hees

**Affiliations:** 1 Department of Pulmonary Diseases, Radboud University Medical Center, 6525 GA Nijmegen, The Netherlandsbram.vandenborst@radboudumc.nl (B.v.d.B.); 2 Department of Physiology, Radboud University Medical Center, 6525 GA Nijmegen, The Netherlandsmaria.hopman@radboudumc.nl (M.T.E.H.); 3 Department of Research and Development, Ciro, 6085 NM Horn, The Netherlands; martijnspruit@ciro-horn.nl (M.A.S.); roymeys@ciro-horn.nl (R.M.); fritsfranssen@ciro-horn.nl (F.M.E.F.); 4 NUTRIM School of Nutrition and Translational Research in Metabolism, Faculty of Health and Life Sciences, Maastricht University, 6229 HX Maastricht, The Netherlands; 5 Department of Respiratory Medicine, Maastricht University Medical Centre (MUMC+), 6229 HX Maastricht, The Netherlands; 6 Department of Pulmonology, Merem Pulmonary Rehabilitation Centre, 1213 VX Hilversum, The Netherlands; pklijn@merem.nl; 7 Department of Pulmonary Medicine, Amsterdam University Medical Centre, 1105 AZ Amsterdam, The Netherlands; 8 Human and Animal Physiology, Wageningen University, 6708 WD Wageningen, The Netherlands; silvie.timmers@wur.nl

**Keywords:** muscle endurance, chronic obstructive pulmonary disease, volitional, non-volitional, quadriceps

## Abstract

Volitional assessment of quadriceps muscle endurance is clinically relevant in patients with chronic obstructive pulmonary disease (COPD). However, studies that determine the construct validity of volitional tests by comparing them to non-volitional measures are lacking. Therefore, the aim of the current study is to evaluate the correlation between volitional and non-volitional quadriceps muscle endurance in patients with COPD. Quadriceps muscle endurance was evaluated in twenty-six patients with COPD. A volitional isometric and a volitional isokinetic protocol were performed on a computerised dynamometer to determine the isometric time and isokinetic work fatigue index, respectively. Non-volitional assessment of quadriceps muscle endurance was evaluated using repetitive electrical stimulations to establish the isometric muscle force decline. Sixteen patients (61 ± 8 years, 63% male, FEV_1_ 47 (32–53)%) performed all three quadriceps endurance tests conforming to pre-defined test criteria. Both volitional isometric time and isokinetic work fatigue index did not significantly correlate with non-volitional muscle force decline (both *p* > 0.05). There was a strong correlation between volitional isometric time and isokinetic work fatigue index (rho = −0.716, *p* = 0.002). To conclude, this study suggests that volitional measures evaluate partly different aspects of quadriceps muscle endurance compared to non-volitional measures. Accordingly, these outcome measures cannot be used interchangeably.

## 1. Introduction

Chronic obstructive pulmonary disease (COPD) is a common and highly relevant health problem that causes airflow limitation and impaired gas exchange [1]. Besides pulmonary complications, patients frequently suffer from extra-pulmonary comorbidities like peripheral muscle dysfunction [2]. The major determinants of impaired functioning of peripheral skeletal muscles comprise muscle fibre atrophy, a slow-to-fast fibre type shift, reduced oxidative enzyme activity, a lower capillary-to-fibre ratio, and reduced mitochondrial density [3,4,5,6,7,8]. During muscular activity, these impairments cause an earlier and higher reliance on glycolytic metabolism, and, in turn, premature lactate production and contractile fatigue [3,4,5,6,7,8]. On a functional level, this can provoke reduced lower-limb muscle strength, endurance, power, or increased fatiguability [3,9,10]. Peripheral muscle dysfunction is a clinically relevant problem as it contributes to exercise intolerance, poor health status, increased healthcare utilisation, and even premature mortality [3]. Therefore, assessment of lower-limb muscle function is essential to identify those patients at risk timely and provide adequate treatment.

Various volitional and non-volitional techniques are available to measure different functional aspects of peripheral muscles in patients with COPD. The choice of a technique is often driven by the functional outcome (i.e., strength, endurance, power, and fatiguability) that the test provides [9]. Muscle endurance is a highly relevant functional outcome in patients with COPD, as it is commonly reduced and closely related to exercise capacity and physical activity in this patient group [11,12,13]. Volitional muscle endurance can be assessed feasibly and reliably using either static (isometric) or dynamic (e.g., isokinetic) contractions [14]. Both isometric and isokinetic quadriceps muscle endurance have been positively associated with muscle oxidative phenotype in patients with COPD [15,16]. However, the validity of volitional quadriceps muscle outcome measures can be affected by external factors like motivation, perceived effort of the patient, and/or dyspnoea sensation [3]. To circumvent the influences of these factors, non-volitional assessment of quadriceps muscle endurance has been applied in experimental settings [17,18]. Recent studies have already reported great reliability and reproducibility of non-volitional assessment of muscle endurance using electrical stimulation and repetitive magnetic stimulation in patients with COPD and in patients with spinal cord injury [19,20,21,22]. However, non-volitional assessment of peripheral muscle function is less practical and more time-consuming in clinical settings compared to volitional assessment. Accordingly, it is important to determine the construct validity of the volitional assessment of quadriceps muscle endurance by comparing it to non-volitional measures. This could further guide clinicians and researchers to make a well-informed decision for an appropriate test protocol to evaluate quadriceps endurance and to improve the interpretation of test results in patients with COPD. The current study aims to evaluate the correlation between volitional isometric and isokinetic quadriceps muscle endurance and non-volitional isometric quadriceps muscle endurance in patients with COPD. Because previous studies have reported strong correlations between volitional and non-volitional assessment of muscle strength in patients with COPD [20,23,24,25], we hypothesised that a strong correlation is also present between volitional and non-volitional outcome measures of quadriceps muscle endurance.

## 2. Materials and Methods

This cross-sectional observational study was conducted between July 2022 and January 2023 at the Radboud University Medical Centre in the Netherlands. Patients with a diagnosis of COPD according to the Global Initiative for COPD report [2] were considered for inclusion at the start of a pulmonary rehabilitation (PR) program. Patients were clinically stable (i.e., no exacerbation and/or hospitalisation within the previous four weeks) and were between 40 and 80 years old. Exclusion criteria were the inability to speak or understand the Dutch language or the presence of musculoskeletal or neurological problems influencing quadriceps muscle function testing. This study was in accordance with the principles of the Declaration of Helsinki and patients provided written informed consent as approved by the local ethical committee (2022-13593). Furthermore, the trial was registered in ClinicalTrials.gov (NCT05427773).

### 2.1. Assessment

Patients were thoroughly and systematically evaluated during an assessment prior to the start of the PR program. Demographics, body mass index, fat-free mass index, and degree of dyspnoea according to the modified Medical Research Council [26] were obtained from electronic patient records. Pulmonary function was assessed using post-bronchodilator spirometry, static lung volumes, and diffusion capacity by single-breath method (MasterScreen PFT/Body; Jaeger, Würzburg, Germany) according to the European Respiratory Society recommendations [27] and was related to predicted normal values [27,28]. Maximal oxygen uptake (absolute and percentage predicted [29]) was determined using a symptom-limited maximal cardiopulmonary exercise test on an electromagnetically braked cycle ergometer (Ergoselect; Ergoline, Bitz, Germany) [30] with breath-by-breath (Vyntus TM CPX; Vyaire Medical, Yorba Linda, United States) analyses. The physical activity of the patient was obtained using the DynaPort MoveMonitor (McRoberts BV, The Hague, The Netherlands), which was worn for seven consecutive days (with a minimum of at least five) [31,32]. Isometric quadriceps maximal voluntary contraction (MVC) of the right leg (or left leg in case of complications) was assessed on a computerised dynamometer (Biodex System 4 Pro, Biodex Medical Systems, Inc., New York, NY, USA). Patients performed 3 maximal unilateral isometric knee extensions for 5 s at a knee angle of 60° flexion, interspersed with 15 s of rest. The coefficient of variation between the 3 contractions did not exceed 10%, and the highest peak torque (Newton-meter) was used [33].

#### 2.1.1. Volitional Quadriceps Muscle Endurance

Volitional isometric and isokinetic quadriceps muscle endurance were assessed in the first week of the PR program. After the isometric protocol, a rest period of at least 30 min was applied before the isokinetic protocol was executed, as described previously [14]. Both protocols were performed on the same computerised dynamometer and on the same leg that was used for the MVC test. Positioning of the patients, including the use of straps, setting up the range of motion, and warm-up protocols were in accordance with previously described and illustrated methods [14]. Strong verbal encouragement was provided during both protocols to motivate maximal effort. Borg dyspnoea and leg fatigue scores were obtained at the start and the end of both endurance protocols [34].

The isometric endurance test protocol was performed by asking the patient to maintain, for as long as possible, an isometric quadriceps contraction representing 60% (with a range of 55–65%) of their individual isometric MVC. The isometric MVC test was repeated in the first week of the PR program when a period of ≥3 months was present between the MVC test and the isometric quadriceps muscle endurance test, or in case an event occurred between both tests that could possibly impair the muscle strength (e.g., exacerbation, illness, prednisone usage). Visual feedback from a computer screen showed the patient’s applied force. The test was ended if the force of the contraction dropped below <50% of the MVC for 3 consecutive seconds. Isometric endurance was defined as the time (s) during which the contraction was maintained ≥50% of the MVC [15].

The isokinetic protocol consisted of 30 contractions at an angular velocity of 90°/s with maximal effort during extension and passive (sub-maximal) flexion [14]. The main outcome was the work fatigue index (%), defined as ($\frac{\mathrm{Work}\;\mathrm{first}\;10\;\mathrm{repetitions}\;-\;\mathrm{Work}\;\mathrm{last}\;10\;\mathrm{repetitions}\;}{\mathrm{Work}\;\mathrm{first}\;10\;\mathrm{repetitions}}\;*\;100\%$) [35]. The following criteria for valid test execution of isokinetic quadriceps muscle testing were applied: completion of all 30 repetitions, peak torque reached within the first 5 repetitions, and presence of work fatigue (positive work fatigue index) [36].

#### 2.1.2. Non-Volitional Quadriceps Muscle Endurance

Non-volitional quadriceps muscle endurance was evaluated using repetitive electrical stimulation at the beginning of the second week of the PR program. The patient was seated on a specially designed chair with the knee flexed at a 60° angle and straps across the chest, pelvis, upper thigh, and ankle of the tested leg (same leg as volitional endurance measurements). Extension forces of the quadriceps were determined using a force transducer (strain gauge) connected to the strap placed around the ankle. First, the isometric MVC was determined by performing at least 3 maximal volitional knee extensions of 3 s, with 1 min of rest in between, until the maximum force was obtained. After placing 2 surface electrodes (Electro-Medical Supplies, Greenham, Wantage, Oxfordshire, UK) on the distal and proximal part of the quadriceps, the current (mA) was determined that corresponded with 40% of the MVC. The 2 min fatigue protocol consisted of 60 electrical stimulations (with a frequency of 30 Hz) of 1 s duration with 1 s of rest in between. Dyspnoea and leg fatigue Borg scores were obtained before and after the fatigue protocol [34]. This fatigue protocol allowed the determination of the muscle force decline (%), defined as ($\frac{\mathrm{Force}\;\mathrm{first}\;3\;\mathrm{stimulations}\;-\;\mathrm{Force}\;\mathrm{last}\;3\;\mathrm{stimulations}}{\mathrm{Force}\;\mathrm{first}\;3\;\mathrm{stimulations}}\;*\;100\%$), by using a custom-made software program to analyse the force records [21,37,38]. Inspection of the raw data was performed to determine whether the patient relaxed fully during the fatigue protocol (i.e., the force between stimulations returned to 0 Newton) and, thus, whether the data could be considered valid.

### 2.2. Statistical Analysis

Data were presented as mean ± SD, median (interquartile range), or number of patients (percentage), as appropriate. A sample size of 16 patients was calculated to be sufficient to obtain a strong correlation (r ≥ 0.8) with precision between 0.50 and 0.93 with a 95% confidence interval, comparable to previous papers on correlations between volitional and non-volitional peripheral muscle strength [20,23,24].

Differences between included and excluded patients were determined using an independent-samples *t*-test, Mann–Whitney U test, or chi-square test, as appropriate. Furthermore, related-samples Friedman’s two-way analysis of variance was used to evaluate differences between Borg scores. Pearson or Spearman correlations were used for normally distributed and skewed data, respectively. A priori, a two-sided significance level was set at <0.05. All statistical analyses were performed using IBM SPSS Statistics 27 (SPSS Inc., Chicago, IL, USA).

## 3. Results

**Figure 1:**
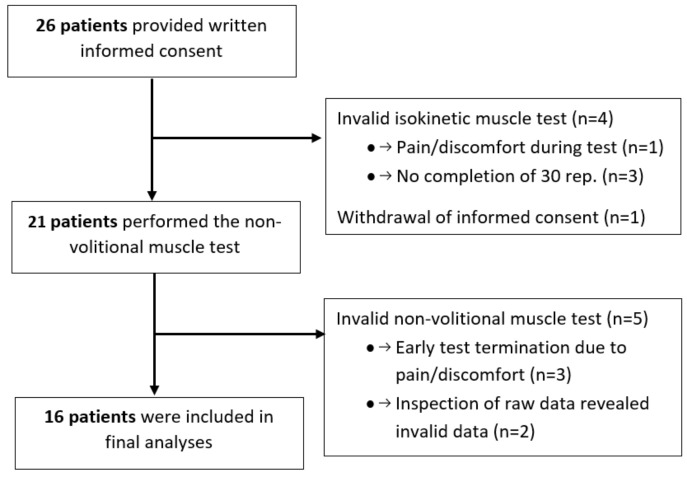
Flowchart of patient inclusion.

A total of 26 patients provided written informed consent, of whom 16 were included in the final analyses. Patients were excluded due to an invalid volitional isokinetic test (n = 4), a withdrawal of informed consent (*n* = 1), or an invalid non-volitional test (*n* = 5) (Figure 1). 

Each patient included in the final analyses showed a decline in force during both the volitional isokinetic and non-volitional isometric endurance tests, indicating fatigue. These patients were 61 ± 8 years old, 38% were male, had a body mass index of 25 ± 5 kg/m^2^, and had poor pulmonary and physical function (Table 1). Besides a significant difference in age (68 ± 6 years vs. 61 ± 8 years, *p* = 0.042), baseline characteristics of the excluded patients were comparable with the included patients.

### 3.1. Volitional and Non-Volitional Quadriceps Muscle Endurance

**Figure 2:**
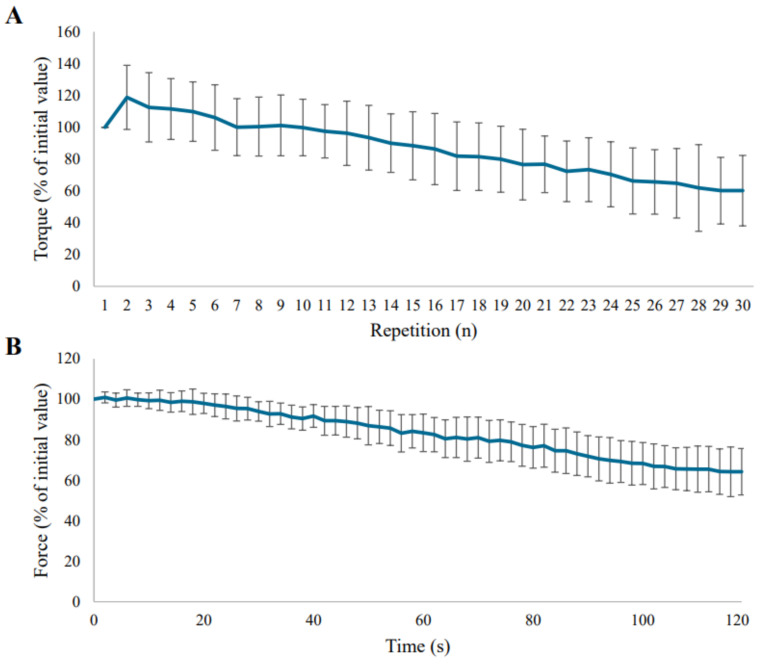
(**A**) Mean torque as percentage of initial value for each repetition during the volitional isokinetic test; (**B**) mean force as percentage of initial value for each electrical stimulation over time during the non-volitional isometric test. The error bars represent the standard deviations.

The median volitional isometric time was 33 (24–36) seconds and the isokinetic work fatigue index was 48 (40–55)%. Figure 2A depicts the mean torque for each of the 30 isokinetic repetitions. The median time between the two volitional endurance tests and non-volitional endurance test was 5 (min–max: 1–12) days. A median non-volitional muscle force decline of 37 (27–43)% was observed. Figure 2B shows the mean force for each of the 60 electrical stimulations over time.

**Figure 3:**
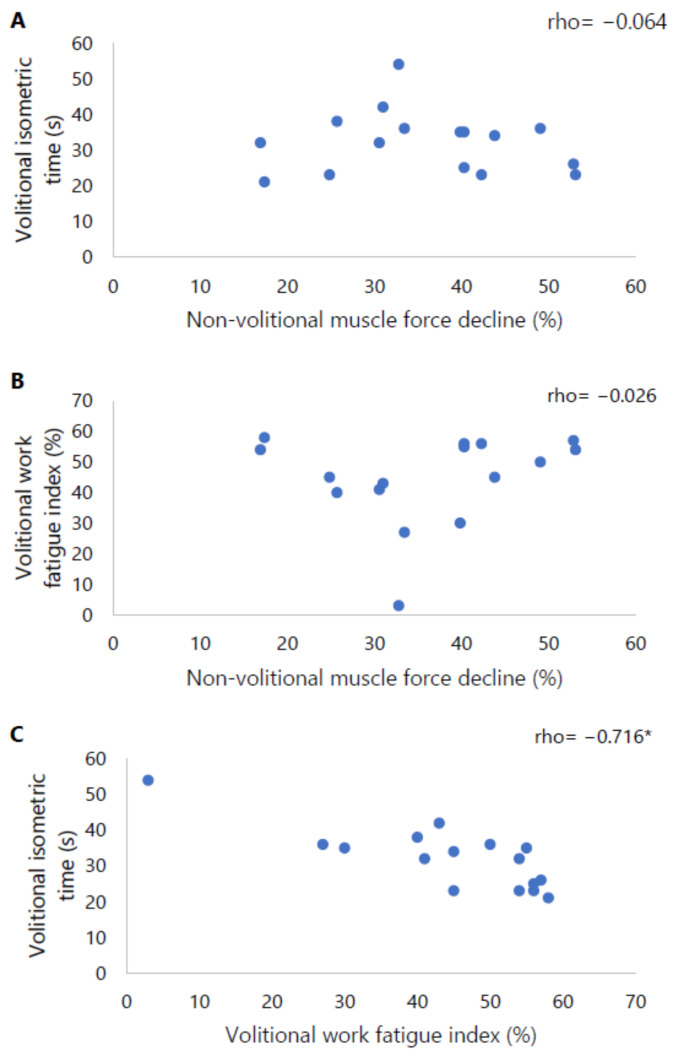
Scatter plots of (**A**) volitional isometric time in seconds with non-volitional muscle force decline as percentage, (**B**) volitional work fatigue index as percentage with non-volitional muscle force decline as percentage, and (**C**) volitional isometric time in seconds with volitional work fatigue index as percentage. * Indicates a significant correlation of *p* < 0.05.

Neither volitionally assessed isometric time nor isokinetic work fatigue index correlated with non-volitionally assessed isometric muscle force decline. A significant relation was observed between the volitionally assessed outcome measures, i.e., isometric time and isokinetic work fatigue index (rho = −0.716, *p* = 0.002) (Figure 3).

#### Borg Scores

Dyspnoea and leg fatigue Borg scores increased significantly during all three endurance tests. No differences were observed in Borg scores at the start of the three tests. At the end of the volitional isokinetic test, patients reported a significantly larger increase in dyspnoea and leg fatigue than at the end of the other two endurance tests (*p* < 0.001) (Table 2). Furthermore, the changes in dyspnoea and leg fatigue Borg scores were significantly related to isokinetic work fatigue index (rho = 0.577, *p* = 0.019 and rho = 0.561, *p* = 0.024, resp.), but not with other volitional or non-volitional endurance outcome measures.

## 4. Discussion

This study is the first to evaluate the relationship between volitional and non-volitional quadriceps muscle endurance within patients with moderate-to-severe COPD. The main finding is that outcome measures of two different volitional tests correlate significantly and strongly, but neither of those show a significant correlation with non-volitionally assessed muscle endurance. The current study, therefore, suggests that volitional tests measure different aspects of quadriceps muscle endurance compared to non-volitional tests.

Volitionally assessed isometric and isokinetic quadriceps muscle endurance were not related to non-volitional electrically stimulated quadriceps muscle endurance in patients with COPD. For maximal quadriceps muscle strength, strong correlations between volitional and non-volitional tests have been reported in patients with COPD [20,23,24]. A possible explanation for this difference can be found in the distinct complexity of the mechanisms that determine performance during muscle strength and endurance tests [39]. Maximal muscle strength is predominantly determined by the quantity and quality of muscle fibres that can be activated, more specifically the number of cross-bridges that can be formed in parallel [40,41,42]. The amount of activated muscle fibres during volitional tests of muscle strength is apparently representative of the number of activated muscle fibres during a stimulation-induced muscle contraction (i.e., non-volitional). The mechanisms that determine muscle endurance are more complex because the ability of the muscle to sustain force for an extended time period is determined by the development of central and peripheral fatigue. Central fatigue is defined as the deficient drive of motor cortical output attenuating performance or even stopping the activity [43]. Peripheral fatigue is described as the reduction in the efficacy of processes at and beyond the neuromuscular junction, such as metabolic and biochemical changes within the muscles [43]. The lack of a strong relation between volitional and non-volitional quadriceps muscle endurance suggests that volitional and non-volitional tests may evaluate different degrees of central and peripheral fatigue [17,18]. Indeed, a study in healthy subjects found only a moderate correlation between central and peripheral fatigue when examined by electromyography analysis during voluntary knee extensions [44]. Central fatigue is proposed to increase the perception of effort and to impair the motivation of the patient. This primarily affects volitional assessment of quadriceps muscle endurance and not, or to a lesser extent, non-volitional assessment, because direct electrical stimulation of the quadriceps muscle precludes the involvement of the central nervous system [43,45,46]. Hence, while the outcome of non-volitional testing is mainly determined by processes of peripheral fatigue, the outcomes of volitional tests are determined by the development of both peripheral and central fatigue. This could explain the lack of a strong relation between outcome measures of volitional and non-volitional assessment of quadriceps muscle endurance. The higher number of determinants of performance during volitional testing compared to non-volitional testing is likely also reflected by the larger standard deviations observed during both tests in Figure 2. Furthermore, it is well known that patients with COPD can develop impairments at the peripheral muscle level, like muscle fibre type shift and reduced oxidative capacity [3,4,5,6,7]. Deficiencies of the central nervous system have also been described in patients with COPD, such as delays in latency periods and deceleration in conduction velocity [47]. Thus, in some patients, neurological deficiencies may also contribute to the development of fatigue. So, the development of central and peripheral fatigue appears to be heterogeneously affected in the population of COPD patients. Therefore, the relation between volitional and non-volitional assessed quadriceps muscle endurance may even be weaker in patients with COPD than in healthy subjects.

The current study showed larger increases in dyspnoea sensation during the volitional isokinetic endurance test than during the non-volitional test. The increase in the severity of dyspnoea was related to a larger work fatigue index but did not correlate with muscle force decline during the non-volitional test. This suggests that the development of dyspnoea affected the outcome of the volitional isokinetic test but not the non-volitional test, which probably also contributed to the lack of correlation between the outcome measures of both tests. Interestingly, the increase in dyspnoea sensation during the volitional isokinetic test was also larger than during the volitional isometric test. This might suggest that the ventilatory demand during the isokinetic test is higher than during the isometric test. Although studies investigating ventilation during isometric testing are currently lacking, it has indeed been shown that pulmonary ventilation is extensively increased during the isokinetic test in patients with COPD [48]. Nevertheless, the current study reported a significant strong correlation between volitional isometric and isokinetic quadriceps muscle endurance. Therefore, despite the difference in applied contraction types and the burden on the ventilatory system, both volitional protocols seem to produce comparable measures of quadriceps muscle endurance.

### 4.1. Strengths and Limitations

To date, there is no consensus yet on the *best* protocol to evaluate quadriceps muscle endurance in patients with COPD in a clinical setting. Therefore, it is highly essential to evaluate the relationship between volitional protocols and non-volitional protocols, as this can provide more insight into the influence of central fatigue (and, thus, the motivation and cooperation of the patient) and dyspnoea during volitional protocols. The current study is the first to evaluate this relation within patients with moderate-to-severe COPD. It is important to emphasise that this study was performed at the start of a PR program, which means that the patients followed their standard clinical care. For example, in the week between the volitional and non-volitional endurance tests patients performed resistance and endurance training sessions, mainly to become familiar with training equipment and exercises. Although it is highly unlikely that these few (median: 1.5) training sessions would already induce substantial training effects on quadriceps muscle endurance, it cannot be excluded that this contributed to the lack of a correlation between volitional and non-volitional endurance tests. Furthermore, it is important to highlight that the current study can only reject the hypothesis that a strong correlation (coefficient of ≥0.8) exists between the volitionally assessed work fatigue index and non-volitionally assessed muscle force decline. Accordingly, the presence of a weak or moderate correlation cannot be excluded and future studies investigating this would require larger sample sizes. Finally, the included patients in the current study are characterised by moderate-to-severe COPD and a high symptom burden. This induced a selection bias as COPD patients with a milder airway obstruction probably experienced less dyspnoea during the isokinetic endurance test and, therefore, also performed this test better than the patients in this study. Accordingly, it could be expected that the severity of the airway obstruction contributed to the lack of correlation between volitional and non-volitional tests.

### 4.2. Clinical Implications and Future Research

The absence of a (strong) correlation between volitional and non-volitional outcome measures of quadriceps endurance in patients of COPD might indicate that these protocols evaluate (partly) different aspects of quadriceps endurance. Therefore, it is suggested that the protocols should not be used interchangeably in both clinical and research settings. In addition, it is essential for the clinician and/or researcher to consider the specific aim of the measurement as well as the tolerance of the patient. Based on the results of this study, it seems that the volitional isokinetic protocol causes the highest sensations of dyspnoea and might, therefore, be less suitable for dyspnoeic patients. The volitional isometric protocol has the highest feasibility and might be recommended for patients with a very low tolerance or when the performance of a second test is not possible and high feasibility is essential. At last, the non-volitional isometric protocol might be more suitable when the aim of the test is to obtain a truer reflection of the peripheral muscle endurance without the interference of motivation and cooperation of the patient, and/or sensations of dyspnoea.

Future research should focus on the reliability of the non-volitional isometric protocol in patients with COPD and/or determine and compare the responsiveness of these protocols following a pulmonary rehabilitation program to further guide clinicians and researchers to make a well-informed choice for a measurement technique.

## 5. Conclusions

This study reports that quadriceps muscle endurance outcome measures of two different volitional tests are strongly related, but neither of those show a strong correlation with non-volitional outcome measures in patients with COPD. This indicates that outcome measures of volitional and non-volitional tests cannot be used interchangeably. Clinicians and researchers should consider, amongst others, the aim of the measurement and the tolerance of the patients when choosing a measurement technique. Future research should investigate the reliability and responsiveness of the non-volitional isometric protocol in patients with COPD to further improve decision making.

## Figures and Tables

**Table 1:** Baseline characteristics of the included patients.

	Included Patients (*n* = 16)
Age (years)	61 ± 8
Gender (male:female)	6:10
BMI (kg/m^2^)	25.2 ± 5.3
FFMI (kg/m^2^)	16.6 ± 2.0
GOLD A/B/E (*n*)	0/8/8
**Lung function**	
FEV_1_ (L)	1.1 (1.0–1.6)
FEV_1_ (% pred)	47 (32–53)
FEV_1_/FVC (%)	37 (30–52)
RV (L)	3.6 (2.8–4.5)
RV (% pred)	176 ± 48
TLC (L)	6.7 (6.0–8.1)
TLC (% pred)	120 ± 14
DLco (mmol/(min·kPa)) *	4.0 ± 1.4
DLco (% pred) *	50 ± 11
mMRC dyspnoea grade	2 (1–3)
**Physical function**	
VO_2_peak (mL/min/kg)	14.4 (12.9–19.9)
VO_2_peak (% pred)	63 (46–77)
Average steps/day	4238 (2713–6811)
MVC (Nm)	108 (98–153)
MVC (% pred)	64 ± 12

Abbreviations: BMI, body mass index; FFMI, fat-free mass index; FEV_1_, forced expiratory volume in one second; FVC, forced vital capacity; GOLD, Global Initiative for Chronic Obstructive Lung Disease; RV, residual volume; TLC, total lung capacity; DLco, lung diffusion capacity for carbon monoxide; VO_2_peak, maximal oxygen consumption; mMRC, modified Medical Research Council peak oxygen consumption; MVC, maximal voluntary contraction. * Indicates a sample size deviant of *n* = 15.

**Table 2:** Dyspnoea and leg fatigue Borg scores for the volitional and non-volitional endurance tests.

	Volitional Isometric Test	Volitional Isokinetic Test	Non-Volitional Isometric Test	*p*-Value
**Borg score dyspnoea** Start End Delta	1 (0–3)	1 (1–3)	1 (1–2)	0.218
	4 (2–6) ^#^	5 (4–7) ^#^	2 (1–3) ^#,^*	<0.001 ^a,b^
	1 (1–3)	4 (2–5)	1 (0–2) *	<0.001 ^a,b^
**Borg score leg fatigue** Start End Delta	1 (0–3)	2 (1–3)	2 (1–3)	0.223
	5 (3–7) ^#^	6 (5–8) ^#^	4 (3–5) ^#,^*	<0.001 ^a,b^
	3 (2–4)	4 (3–5)	2 (0–3) *	<0.001 ^b^

^a^ Indicates a significant post hoc difference between the volitional isometric test and the volitional isokinetic test. ^b^ Indicates a significant post hoc difference between the volitional isokinetic test and the non-volitional isometric test. ^#^ Indicates a significant change in Borg score between the start and end. * Indicates a sample size deviant of *n* = 15.

## Data Availability

The dataset generated during and/or analysed during the current study is available from the corresponding author upon reasonable request.
